# Effect of apical root resection, orthodontic extrusion, and surgical crown lengthening on load capability

**DOI:** 10.1007/s00784-023-05057-4

**Published:** 2023-05-10

**Authors:** M. Naumann, U. Adali, M. Rosentritt, A. Happe, R. Frankenberger, G. Sterzenbach

**Affiliations:** 1 Department of Prosthodontics, Geriatric Dentistry and Craniomandibular, Disorders, Charité – Universitätsmedizin Berlin, Aßmannshauser Str. 4-6, 14197 Berlin, Germany; 2 Department of Prosthetic Dentistry, Regensburg University Medical Center, Regensburg, Germany; 3 Department of Prosthetic Dentistry, University of Ulm, Ulm, Germany; 4 Department of Operative Dentistry and Endodontology, University of Marburg, Georg-Voigt-Str. 3, 35039 Marburg, Germany

**Keywords:** Dental caries, Dental cements, Dental restoration failure, Dental restoration, Permanent, Tooth, Nonvital, Resin cements, Nonvital tooth, Nonvital teeth, Endodontically treated teeth, Endodontically treated tooth

## Abstract

**Objectives:**

This study aims to investigate the load-to-fracture of procedures changing crown-to-root ratio (*R*_CRR_) aimed to restore severely damaged upper central incisors to avoid tooth extraction compared to implant placement. There is no evidence on load capability after apical root resection (AR), orthodontic extrusion (OE), and surgical crown lengthening (SCL) in respect to *R*_CRR_, respectively.

**Material and methods:**

Human maxillary central incisors were endodontically treated, decoronated, and divided into 4 groups (*n* = 48). The following specimen preparation was performed: (I) adhesive core-and-post build-up (control), (II) as (I) and 2 mm apical root resection (AR), (III) before adhesive core-and-post build-up teeth were shortened 2 mm coronally (OE) (IV) as (I), but specimens were embedded 4 mm instead of 2 mm below the CEJ (SCL), group (V) implant-borne restoration with individual all-ceramic abutments (*n* = 12; ∅4.1/l = 12 mm) (IBR). All specimens received all-ceramic crowns, thermo-mechanical (TML), and subsequent linear loading (LL) until failure. *R*_CRR_ were calculated and log-rank, Kruskal–Wallis, Mann–Whitney *U*, ANOVA, and chi-square tests applied (*p* = 0.05).

**Results:**

Fracture loads after subsequent LL differed significantly (*p* = 0.001) between groups, while implants showed the highest values. *F*_max_ median (min/max) were as follows: (I) 252 (204/542), (II) 293 (243/443), (III) 253 (183/371), (IV) 195 (140/274), and (V) 446 (370/539). Pair-wise comparison showed significant differences (*p* = 0.001) between group I/IV and group V, I, and IV (*p* = 0.045), II and IV (*p* = 0.001), and III compared to IV (*p* = 0.033), respectively. *R*_CRR_ below 1 significantly increased load capability compared to *R*_CRR_ = 1.

**Conclusions:**

OE appears to preferably ensure biomechanical stability of teeth that are endodontically treated and receive core-and-post and crown placement compared to SCL. AR has no adverse biomechanical impact. *R*_CRR_ < 1 is biomechanically beneficial.

**Clinical relevance:**

For endodontically treated and restored teeth, orthodontic extrusion should be preferred compared to surgical crown lengthening prior single-crown restoration. As orthodontic extrusion, apical root resection has no adverse effect on load capability. Single-crown implant-borne restorations are most load capable.

## Introduction

Dental implants have become a viable alternative of tooth retention by complex restorative treatments of deeply destroyed teeth. Some authors consider endodontically treated teeth (ETT) as inferior with regard to reliability and cost-effectiveness compared to vital teeth [1] or implants [2]. Systematically reviewed data over 3 to 25 years suggest that the survival rates after endodontic treatment followed by coronal restoration were ~ 81% to 100% [3]. Three systematic reviews confirmed that teeth with endodontic treatment and implant-supported restorations have similar long-term survival [3–5]. It is therefore not surprising that dentists’ opinions on what treatment to recommend vary widely [6]. Concerns regarding aesthetic challenges with implant therapy [7] and in particular biological complications such as peri-implantitis [8] may favour tooth retention.

The preparation of a 2-mm ferrule is considered paramount for long-term success of post-and-core crown restorations [9]. However, in the anterior maxillary region, the distance between the apical defect extension and the alveolar crest is usually insufficient to allow for a ferrule in addition to biologic width [10, 11]. Therefore, additional measures such as surgical crown lengthening or orthodontic or surgical extrusion may be necessary to ensure a ferrule of at least 2 mm height [12]. Both are similar techniques of the so-called forced eruption. They differ only technically in the way extrusion is operated. Hence, in the following, only the term “extrusion” will be used. Evidence on the biomechanical effect of extrusion is scarce. Furthermore, apical pathology in ETT may necessitate apical root resection. All these measures will inevitably result in alterations of the crown-to-root ratio (*R*_CRR_). *R*_CRR_ is a parameter that is traditionally considered as biomechanically relevant in terms of load capability and ultimately tooth survival, although its impact was not studied before.

Hence, the aim of the present study was to evaluate the ex vivo survival and load capability of severely damaged, endodontically treated, post-and-core, and all-ceramic crown-restored maxillary central incisors compared to implant-borne restoration following dynamic and subsequent linear loading.

The following null hypotheses were stated:

(I) There is no difference between tooth-based restorations following simulated extrusion, apical root resection, and surgical crown lengthening, and implant-borne restorations.
(II) Crown-to-root ratio (*R*_CRR_) has no impact on load capability and fracture patterns.

## Material and methods

### Specimen pre-treatment

Human maxillary incisors were selected from a tooth bank and stored at room temperature in a 0.5% chloramine solution. To ensure the use of teeth of comparable dimension within the groups, mesio-distal (MD) and facial–lingual (FL) dimensions were measured at the level of the cemento-enamel junction (CEJ). Tooth size was calculated as the product of MD × FL. Teeth with extremely short (< 12.3 mm) or long root length (> 16.7 mm) were excluded. Specimens were randomly distributed into 4 groups (*n* = 12) by means of a ten-digit random table to either no change to crown-to-root ratio (control, group I), apical root resection (group II), extrusion (group III), and surgical crown lengthening (group IV). The crowns of teeth in groups I and II were cut 2 mm coronally to the CEJ and in groups III and IV at CEJ level. Root canals were enlarged using the X-Smart (Dentsply DeTrey, Konstanz, Germany) and NiTi-files to size F2 (Protaper, Dentsply DeTrey) and rinsed with 3% sodium hypochlorite. Root canals were filled by corresponding size F2 of gutta-percha (Protaper, Dentsply DeTrey) and sealer (AH 26 Plus Jet, Dentsply DeTrey).

The roots of the specimens of groups I and II were blocked out with wax 2 mm and specimens of group III and IV 4 mm below the CEJ. To imitate a periodontium and physiological tooth mobility, roots of the teeth were covered with a layer of silicone (Mollosil Plus, Detax, Ettlingen, Germany) as described elsewhere [13]. All teeth and implants (group V, *n* = 12) were embedded in acrylic resin (Technovit 4004, Kulzer, Wehrheim, Germany). To prevent overheating, the teeth were immersed in water for 5 min during resin polymerization.

### Tooth-based restoration, groups I–IV

Post-space preparation was performed 8 mm within the root canal in one sequence as described by the manufacturer. All restorative steps were performed using the Dentsply Core & Post System (CTS, Dentsply DeTrey).

**Fig. 1:**
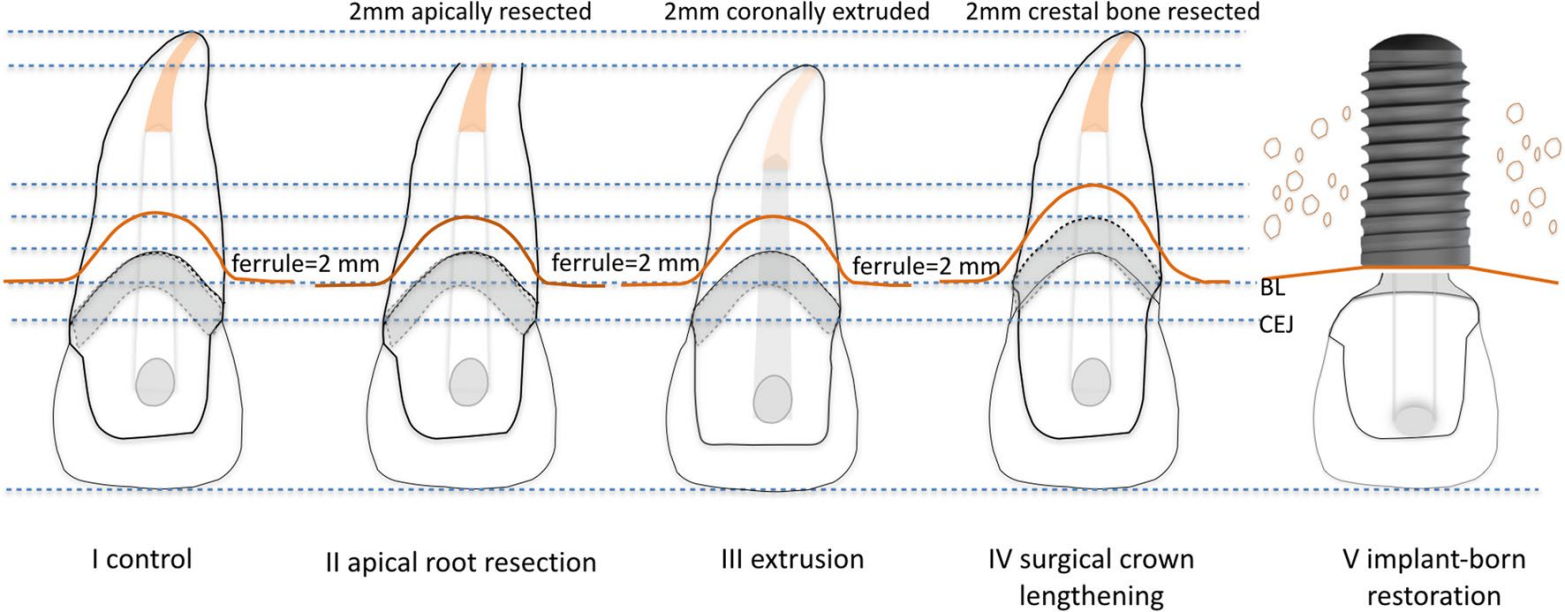
Experimental groups with relative root and crown length, ferrule height and simulated bone level

**Fig. 2:**
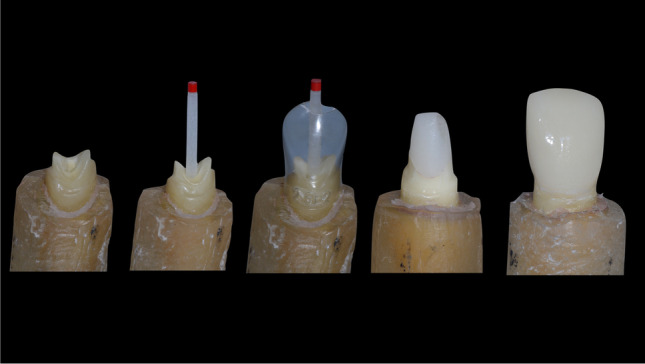
Restorative steps of post-and-core restoration from post to crown placement

The etch-and-rinse and bonding procedure was performed according to the manufacturer’s instruction. The root canal and the coronal tooth surface were etched with 36% phosphoric acid (Conditioner 36, Dentsply DeTrey) for 15 s. After water rinsing and air-drying, the adhesive was applied and left for 20 s (XP Bond, Self-cure Activator, Dentsply DeTrey, 1:1 ratio, mixed for 2 s). A glass fibre post (size 2 (red), Ø 1.25 mm, X-Post, CTS, Dentsply DeTrey) was treated with adhesive and was luted with core build-up material in a staged procedure using a dual curing core-and-post composite (Core-X™ flow, Dentsply DeTrey). The core was built up by means of a strip crown (upper central incisor, Frasaco, Tettnang, Germany) and polymerized from the incisal, palatal, and facial aspect for 20 s each. All teeth were prepared with a circumferential 0.8 mm shoulder and 6° convergence angle to meet all-ceramic crown requirements. To achieve an equal crown length, the core build-ups were similar in length for group I to III (4 mm) and for group IV (6 mm). The margin was located 2 mm below the core build-up in dentin to ensure proper ferrule design. Specimens were scanned with an intraoral scanner (Trios, 3Shape, Kopenhagen, Dänemark); models were milled out of polyurethan. Crowns were constructed digitally (Dental Designer, 3Shape, Copenhagen, Denmark), subsequently milled in wax (Organical Multi, R + K CAD/CAM, Berlin, Germany), transferred to lithium disilicate (IPS e.max, Ivoclar Vivadent, Schaan, Germany), and glazed (IPS e.max Ceram Glaze, Ivoclar Vivadent). Crown width was 2 mm incisally, 1.5 mm in the middle, and 0.8 mm at the preparation margin. Crown height was 8 mm, except in group IV (10 mm) (Figs. 1 and 2).

### Implant-based restoration, group V

Dental implants (length 12 mm, diameter 4.1 mm, Straumann Bone Level, Freiburg, Germany) were restored with titanium alloy base (RC Variobase Abutment, diameter 4.5 mm, length 3.5 mm, Straumann, Germany) with lithium disilicate abutments (IPS e.max, Ivoclar Vivadent, Schaan, Liechtenstein). Twelve identical lithium disilicate abutments were modelled in wax (Dental Designer, 3Shape, Copenhagen), milled (Organical Multi, R + K CAD/CAM, Berlin), and transferred in lithium disilicate (IPS e.max, Ivoclar Vivadent). Abutment measures were equivalent to cores of group I. Abutments were luted on the alloy bases with self-adhesive luting composites (IPS E.max Abutment Solution Cem Kit, Ivoclar Vivadent). Abutments were screwed in with 35Ncm. Crowns were etched 20 s with fluoric acid (Vita Ceramics Etch, Vita, Bad Säckingen, Germany), cleaned with water and isopropanol, silanized (Monobond Plus, Ivoclar Vivadent, Schaan) for 60 s, and self-adhesively luted (SmartCem Dentsply DeTrey). Final light curing was performed for 20 s from each restoration side.

### Loading protocol

**Fig. 3:**
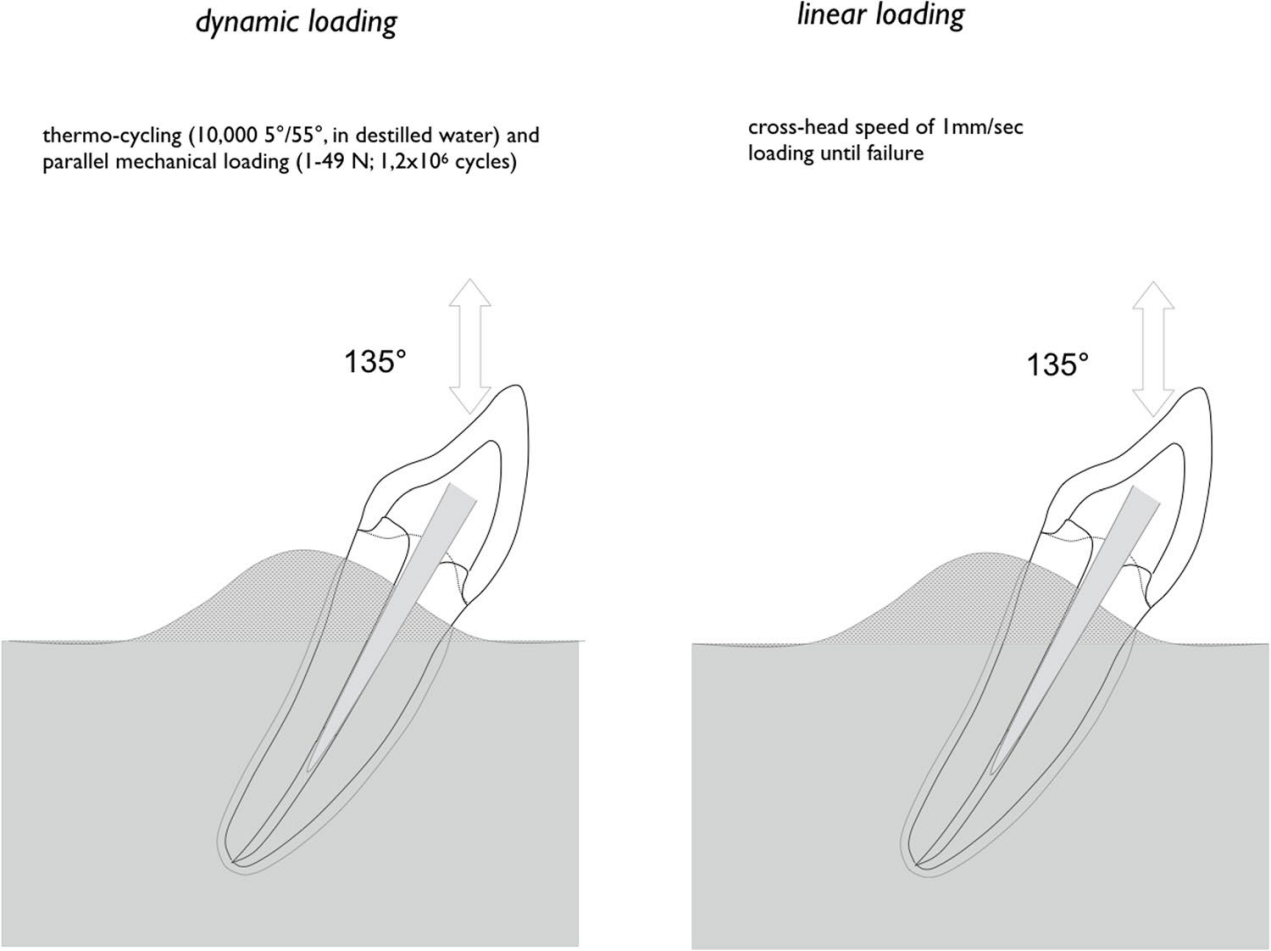
Examples of typical failure modes per experimental group

Thermal and mechanical loading (TML) was performed (parameters: 6000 thermal cycles, 5 °C/ 55 °C, 2 min each cycle; dist. water; 1.2 × 10^6^ mastication cycles with 50N) to simulate 5 years of clinical service [14]. The restorations were loaded under 135°, 3 mm below the incisal edge, on the palatal surface of the crown. After TCML, tooth mobility was measured three times for each specimen by means of a Periotest device perpendicular to tooth and implant axis (Periotest Classic, Medizintechnik Gulden, Germany). Specimens were statically loaded in a universal testing machine (Zwick 1446, Zwick, Ulm, Germany; v = 1 mm/min) until failure. Failure detection was set at a 10% loss of the maximum applied force. A 0.3-mm-thick tin foil was positioned between the steel piston and the palatal crown surface to reduce excessive stress concentrations (Fig. 3).

### Calculation of crown-to-root ratio (R_CRR_)

The length of the prosthetic crown *L*_C_ was defined with 8 mm for groups I to III and 10 mm for group IV. The distance crown margin to crestal alveolar bone was 2 mm in order to simulate biologic width (*L*_BW_). The sum of *L*_C_ + *L*_BW_ was defined as effective crown height *C*_E_. The root length *L*_R_ is the distance from the apex to the CEJ. The effective root length *R*_E_ is defined as length of the root *L*_R_ within alveolar bone [15]. In groups II, III, and IV, *R*_E_ was 2 mm smaller than *L*_R_ due to simulated apical root resection, extrusion, or crown lengthening, respectively (*R*_E_ = L_R_ − 2 mm). In groups II and III, the effective crown height *C*_E_ was equivalent to that of group I. For group IV, *C*_E_ of group IV was calculated from *L*_C_ + *L*_BW_ + 2 mm of crestal bone resection.

### Statistical analysis

The number of cycles until failure during dynamic loading (TML) was compared with log-rank statistics. Non-parametric Kruskal–Wallis and Mann–Whitney *U* test as post hoc were applied to determine differences between group median values of the crown-to-root ratios (*R*_CRR_), as well as maximum load capability *F*_max_ after linear loading. Differences in the frequency of the failure modes between the groups were evaluated by chi-square tests. Data were pooled and categorized into four patterns: crown fracture, crown fracture with additional core build-up loosening, and fracture at the coronal or middle third of the root (root fracture). Subsequently, failures were classified as “restorable” (crown fracture, crown fracture with core loosening) and “not restorable” (i.e. root fracture). We conducted both a complete case analysis of maximum load capability, i.e. excluding specimens that failed during TML for analysis of *F*_max_, and a sensitivity analysis assigning those specimens that failed *F*_max_ = 0. All statistical tests were two-sided at α = 0.05.

## Results

One early failure in each of the tooth-based restoration groups (groups I–IV) during TML was observed. Early failures in group I, II, and IV were catastrophic root fractures and in group IV a restorable crown fracture. Two implant-supported restorations failed during TML, comprising one restorable abutment/crown fracture and one restorable crown fracture. The early failure rate during TML did not differ significantly between groups (*p* = 0.948).

**Fig. 4:**
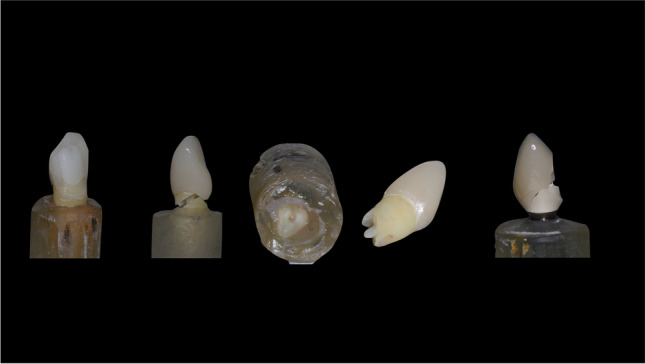
Scatter plot of crown-to-root ratios of experimental groups and respective statistical group differences

Specimens that failed early in TML were excluded from further analysis. Median fracture load ranged from 195 N (group IV) to 446 N (group V), with statistically significant differences between groups (*p* = 0.001). The same is also true (*p* = 0.002) when early TML failures were included in analysis and were assigned a static load of “*F*_max_ = 0”. The pair-wise comparison showed significant differences between all tooth-based groups (I–IV) and the implant group V (*p* = 0.001) and surgical crown lengthening group IV compared to group I (*p* = 0.045), group II apical root resection (*p* = 0.001), and group III extrusion (*p* = 0.033), respectively. Inclusion of specimens of “*F*_max_ = 0” did also reveal significant differences between the implant and all other groups (*p* = 0.002). Neither the comparison of fracture pattern frequency (p = 0.649) nor analysis of clinical judgement in “restorable” vs. “catastrophic” failures revealed statistically significant differences (*p* = 0.172) between the tooth-based groups. Due to its completely different nature, implant-borne restoration was not included in this analysis. All implants were capable to be re-restored (Fig. 4).

Calculated crown-to-root height ratios *R*_CRR_ differed significantly among the experimental groups except for group II compared to group III. In both experimental groups, the root length was reduced 2 mm for different reasons, i.e. apical resection or coronal extrusion. The most unfavourable ratios were calculated for group IV. The latter does show a 25% worse *R*_CRR_ than the control. A *R*_CRR_ below 1 appears to be favourable, and there is a trend that the higher *R*_CRR_ the lower load capability.

**Fig. 5:**
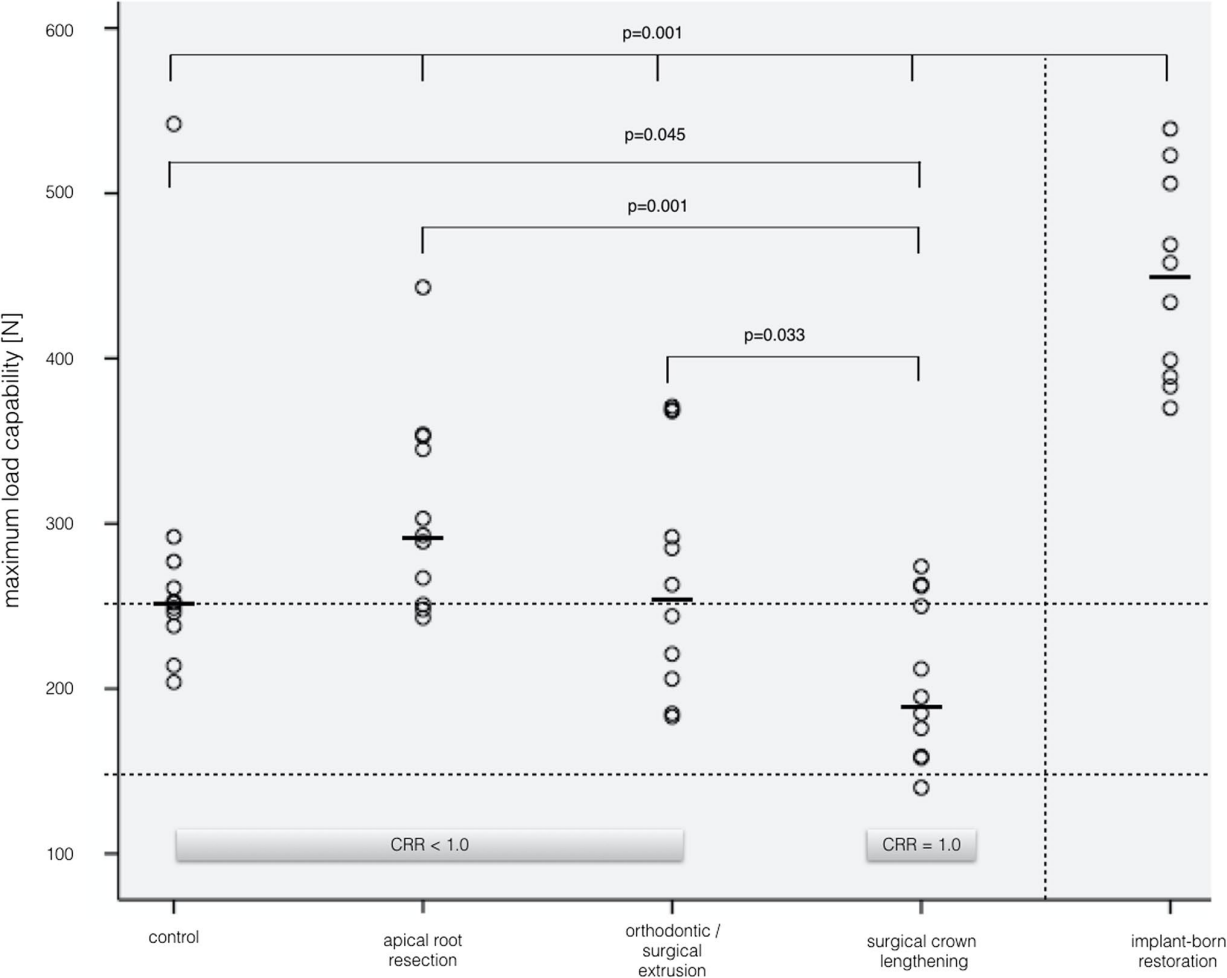
Scatter plot of experimental group load values after TML and subsequent linear loading with *p* values of respective statistically significant differences

**Fig. 6:**
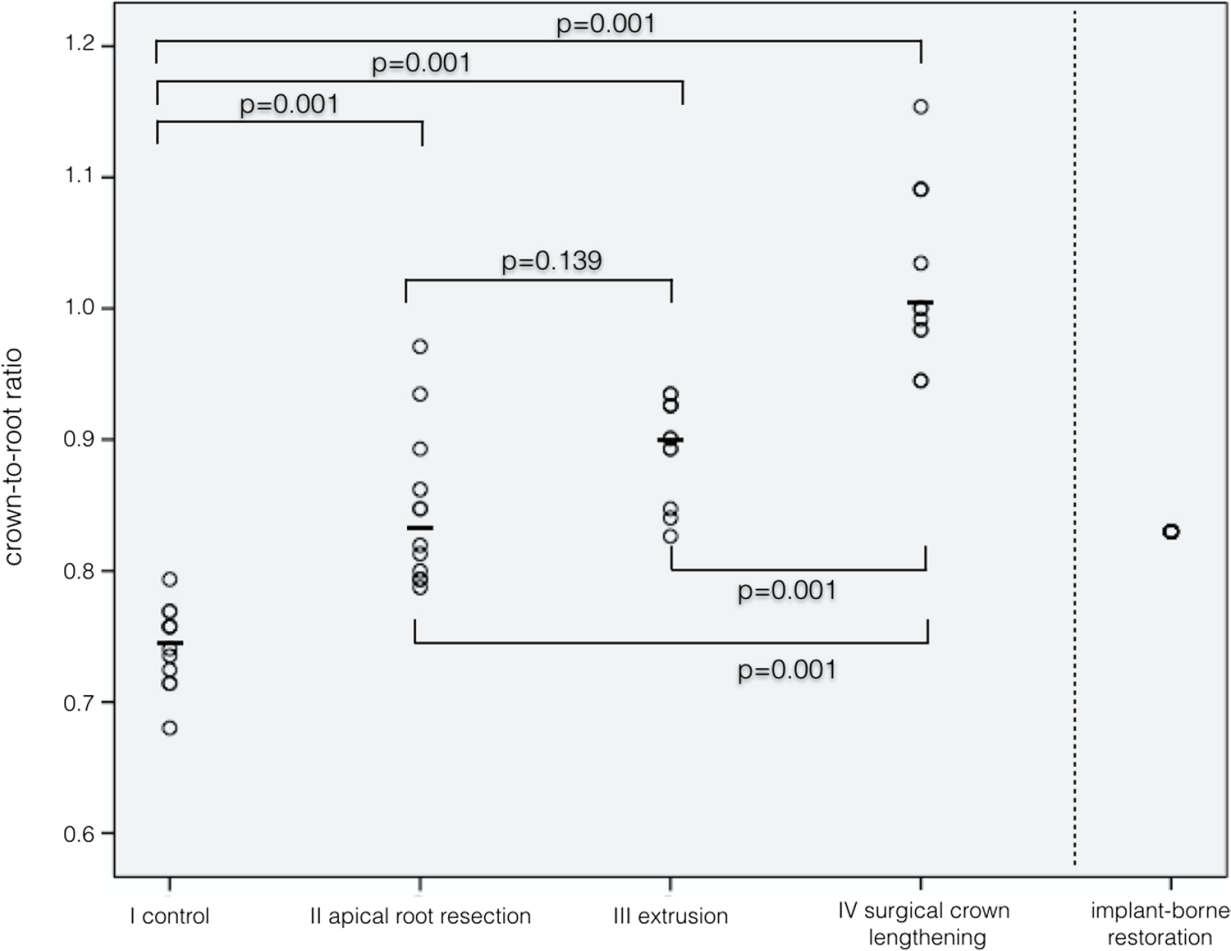
Crown-to-root ratio

Table 1 provides detailed information about specimen mobility, effective root length, crown-to-root ratio, early failures during TML, load capability values, failure patterns and frequency, and their respective clinical judgement, i.e. when a re-restoration of the teeth would be clinical possible. Figure 5 displays the scatterplot of *R*_CRR_ and Fig. 6 of maximum load capability after linear load testing (median values as bold line).

**Table 1:** Specimen details, number of preliminary failures during TML, failure type, and mean values for the load capability in *N* of linear load testing after TML

#	Group	*N*	Periotest value (range)	Effective root length (*R*_E_) Mean (SD)	Crown-to-root ratio Mean (SD)	Early TCML failure [*n*]	Linear loading *F*_max_ Median (min./max.) [*N*]	Restorable failure ^§^ [*n*]	Catastrophic failure * [*n*]
I	Control	12	6 (2.45)	13.48 (0.57)	0.74 (0.03)	1*	252 (204/542)	9	3
II	Apical root resection	12	5.82 (1.6)	11.86 (0.79)	0.85 (0.06)	1*	293 (243/443)	4	8
III	Orthodontic extrusion	12	6.8 (2.44)	11.18 (0.49)	0.90 (0.04)	1*	253 (183/371)	6	6
IV	Surgical crown lengthening	12	5.91 (3.3)	11.74 (0.74)	1.03 (0.07)	1^♯^	195 (140/274)	8	4
V	Implant	12	2.5 (1.43)	12	0.83	2^§^	446 (370/539)	12	0

^**§**^Crown and abutment fracture; *catastrophic root fractures in the coronal or middle third; ^♯^restorable crown fracture

## Discussion

To the best of our knowledge, this is the first study investigating the effect of treatments, which alter crown-to-root ratio *R*_CRR_. Apical root resection, extrusion, and surgical crown lengthening were investigated in severely damaged, endodontically treated, post-and-core restored teeth. The respective treatment modalities show their effect due to relative location of CEJ, apex, root canal filling, post-length or post-tip positon, and bone height. Treatment modalities were compared to implant-borne restorations. Tooth-based restorations are significantly less load capable than implant-borne restorations. Surgical crown lengthened teeth showed lowest load values. *R*_CRR_ for this group was significantly higher. Failure pattern frequency and clinical judgements were equally distributed among tooth-based groups. All implant-borne restoration failed on restoration level and was the most load capable. All null hypotheses were rejected.

Dynamic loading combine thermal and mechanical loading (TML) aimed to provide sufficient prognosis of clinical failure [14]. TML includes fatigue phenomena and is of utmost importance to increase the predictive power of in vitro data in regard to clinical survival of restorations [16]. Fatigue failures are defined as fractures of a material caused by cyclic or repeated subcritical loads aimed to simulate the fatigue phenomenon, attempting to avoid false conclusions based on application of compressive loads only [17]. It may also help to exclude catastrophic clinical failure [14]. The angle of 135° refers to a class-I anterior dentition [18]. Physiologic tooth mobility was always ensured [19].

Upper central incisors were chosen, since they are the most frequently crowned type of tooth [20]. Upper front teeth are regarded as “high-risk area” for mechanical failures caused by fatigue [21], since the amount of shear forces is higher compared to the posterior region. The bonding, core build-up, and post materials used perform well under in vitro conditions [22]. Limitations of this in vitro assay for clinical conclusions may be expected due to the limited but compared to most in vitro studies increased number of specimens [18].

The terms “effective crown height” and “effective root length” were adopted elsewhere [15]. Its ratio is the physical relationship between the portion of the tooth within the alveolar bone compared with the portion above, as determined radiographically [23]. Crown-to-root ratio *R*_*CRR*_ is commonly used in prosthodontics as variable to evaluate an abutment tooth [24, 25]. Vague terms as “favourable”, “appropriate”, “unfavourable”, “poor”, and “unsatisfactory” are used [26]. A longitudinal practice-based study on 236 clasp-retained removable partial dentures found a significant risk of abutment failure when *R*_*CRR*_ is > 1 [24]. For 100 treated periodontal patients, under maintenance for 5 years was found that an “unfavourable” *R*_CRR_ does reduce initial tooth prognosis [27]. Abutment teeth in patients with chronic periodontitis *R*_CRR_ of 1 were recommended as “restorative factor”, when post-and-core restorations are planned, but still judged as “problematic” [28]. The latter is supported by the present study. A *R*_CRR_ of 1 for surgical crown lengthening revealed lower load capability than *R*_CRR_ values < 1. However, this concept of *R*_CRR_ associated with natural teeth does not apply to implant-borne restorations [29]. Since convincing data are scarce that these treatment approaches including endodontic treatment, post-and-core, and final crown restoration can be regarded as equivalent to implant therapy regarding load capability, the latter was also included as alternative treatment option. Clinically, it is a frequently discussed challenging dilemma between tooth retention and implant placement [4] with an impact on the treatment decision of dentists [2]. From a cost–benefit perspective, implant placement owned a role as third-line intervention, when endodontic treatment and re-treatment failed [30]. The present study shows that even early mechanical implant failure may occur and provoke additional cost.

Evidence on the biomechanical effect of apical root resection is scarce. A 5-year longitudinal clinical study on root resection outcome reported marginal bone level as one of only two significant outcome predictors [31]. Only a weak biomechanical influence of apical root resection was found compared with that of periodontal bone loss as caused by surgical crown lengthening [32]. This finding is supported by our observation. When apical root resection and extrusion are performed, effective root length is reduced for both, but the coronal lever arm, i.e. effective crown length, remains constant. Thus, *R*_*CRR*_ is similar for apical root resection and extrusion and decreased up to 15% compared to control. However, it was found that reduced effective root length in apical root resected or extruded teeth is not as harmful as for crestal bone loss due to surgical crown lengthening, since the maximum stress from mastication concentrates on the cervical area. Minimum stress was found on the apical third [33]. *S*tresses increase 6 mm below CEJ up to 10 times compared to normal bone height [34]. Combined tension and compression forces result in oblique crack propagation from the palatal CEJ to the apical third quarter of the facial aspect, and fracture occurs as observed in this study. Thus, it was assumed that *R*_CRR_ is not valid in evaluation of the prosthodontics prognosis of root resected teeth [35].

Surgical crown lengthening increases effective crown height [15]. This clinical situation is biomechanically comparable to reduced but healthy periodontium [36]. The periodontium does have a central role in stress distribution and stress reduction [37]. The widening of periodontal ligament increases tooth mobility as seen clinically and can be seen as “self-defensive effect” to avoid fracture [34]. The effective *R*_CRR_ in this study is increased by remarkable 25% compared to control. This went along with significant decreased load capability compared to all other groups. This is in accordance with in vitro results on statically loaded 2nd premolar analogues where surgical crown lengthening had “unexpectedly significant mechanical costs” [15]. Another in vitro study on maxillary central incisors with simulated bone loss found that loss of 25% and 50% bone height had a markedly decreased load capability in a “dose”-dependent way [38]. Alveolar bone loss leads to increased risk for fracture as shown in a longitudinal clinical study. It was found that ETT in patients with advanced periodontal disease tend to fracture more frequently, primarily when endodontic posts were present [39]. Thus, results of the present study show that there is an adverse biomechanically impact of surgical crown lengthening which should be carefully considered within treatment planning. Apical root resection and extrusion are less problematic. These findings should be verified in clinical pilot studies to enable subsequent power analysis for clinical trials with appropriate sample.

## Conclusion

Within the limitations of this ex vivo study, it can be concluded that surgical crown lengthening has an adverse biomechanical impact on load capability of crown-restored, severely damaged post-supported ETT. Therefore, extrusion appears to be preferable to ensure a 2-mm ferrule, but prospective studies would be necessary to verify their results and to assess whether they are clinically significant. Apical root resection has no adverse biomechanical impact. A *R*_CRR_ < 1 appears beneficial. Implant-borne restorations are more load capable, but mechanical failures during subcritical loading may occur.
